# Rubbertown Next Generation Emissions Measurement Demonstration Project

**DOI:** 10.3390/ijerph16112041

**Published:** 2019-06-08

**Authors:** Eben Thoma, Ingrid George, Rachelle Duvall, Tai Wu, Donald Whitaker, Karen Oliver, Shaibal Mukerjee, Halley Brantley, Jane Spann, Tiereny Bell, Njeri Carlton-Carew, Parikshit Deshmukh, Jacob Cansler, Tamira Cousett, Wei Tang, Andrea Cooley, Kyle Zimmerman, Billy DeWitt, Bryan Paris

**Affiliations:** 1 U.S. Environmental Protection Agency, Office of Research and Development, National Risk Management Research Laboratory, 109 TW Alexander Dr., RTP, NC 27711, USA; George.Ingrid@epa.gov (I.G.); Duvall.Rachelle@epa.gov (R.D.); Wu.Tai@epa.gov (T.W.); 2 U.S. Environmental Protection Agency, Office of Research and Development, National Exposure Research Laboratory, 109 TW Alexander Dr., RTP, NC 27711, USA; Whitaker.Donald@epa.gov (D.W.); Oliver.Karen@epa.gov (K.O.); Mukerjee.Shaibal@epa.gov (S.M.); 3 Former Oak Ridge Institute of Science and Engineering Fellow with EPA ORD, now with NC State University, 2311 Stinson Dr. Raleigh, NC 27695, USA; hlbrantl@ncsu.edu; 4 U.S. Environmental Protection Agency, Region 4, 61 Forsyth St. SW, Atlanta, GA 30303, USA; Spann.Jane@epa.gov (J.S.); Bell.Tiereny@epa.gov (T.B.); Carlton-Carew.Njeri@epa.gov (N.C.-C.); 5 Jacobs Technology Inc., 109 TW Alexander Dr., RTP, NC 27711, USA; Parikshit.Deshmukh@jacobs.com (P.D.); Jacob.Cansler@jacobs.com (J.C.); Tamira.Cousett@jacobs.com (T.C.); 6 Applied Research Associates Inc., 109 TW Alexander Dr., RTP, NC 27711, USA; WTang@ara.com; 7 City of Louisville Metro Air Pollution Control District, 701 W. Ormsby Ave. Ste. 303, Louisville, KY 40203, USA; Andrea.Cooley@louisvilleky.gov (A.C.); Kyle.Zimmerman@louisvilleky.gov (K.Z.); Billy.DeWitt@louisvilleky.gov (B.D.); Bryan.Paris@louisvilleky.gov (B.P.)

**Keywords:** fugitive emission, fenceline monitoring, NGEM, 1,3-butadiene, method 325, auto-GC

## Abstract

Industrial facilities and other sources can emit air pollutants from fugitive leaks, process malfunctions and area sources that can be difficult to understand and to manage. Next generation emissions measurement (NGEM) approaches executed near facilities are enabling new ways to assess these sources and their impacts to nearby populations. This paper describes complementary uses of emerging NGEM systems in a Louisville, KY industrial district (Rubbertown), focusing on an important area air toxic, 1,3-butadiene. Over a one-year deployment starting in September 2017, two-week average passive samplers (PSs) at 11 sites showed both geospatial and temporal trends. At 0.24 ppbv annual average 1,3-butadiene concentration, a group of PSs located near facility fence lines was elevated compared to a PS group located in the community and upwind from facilities (0.07 ppbv average). Two elevated PS periods capturing emission events were examined using time-resolved NGEM approaches as case studies. In one event a 1.18 ppbv PS reading was found to be relatively localized and was caused by a multiday emission from a yet to be identified, non-facility source. In the other event, the airshed was more broadly impacted with PS concentrations ranging from 0.71 ppbv for the near-facility group to 0.46 ppbv for the community group. This case was likely influenced by a known emission event at an industrial facility. For both case studies, air pollutant and wind data from prototype NGEM systems were combined with source location models to inform the emission events. This research illustrates the power of applying NGEM approaches to improve both the understanding of emissions near sources and knowledge of impacts to near-source communities.

## 1. Introduction

Refineries, chemical plants, energy production sites, waste facilities and other industries and commercial operations can emit air pollutants and odorous compounds from a variety of sources. Using established air quality models, the potential public health impact of well-characterized industrial sources can be confidently understood and communicated. Industrial sources that have a known emission point (e.g., stack or vent), a predictable temporal profile, and accurate emissions test data or engineering calculations (e.g., fuel-based or other emission factors) are in this category. Air pollutant emissions can also originate from more complex industrial sources such as fugitive equipment leaks, waste ponds and sewers, process startups and malfunctions, and improperly controlled operations. Broadly labeled as stochastic industrial sources (SISs), this category of emissions is generally less well understood, with air shed and environmental impacts that are more difficult to model.

SIS emissions can be spatially distributed and temporally variable or randomly occurring, with a source’s location or even its existence sometimes unknown [1,2,3]. Some SISs, such as open processing, landfills, and treatment/storage ponds, are extended in area with emissions that are determined in part by meteorological conditions [4,5]. Since many SISs are located near ground level, near-source exposure potential and odor nuisance issues are exacerbated by unfavorable atmospheric conditions at the time of emission [6]. A key to achieving better understanding and management of SISs lies in development of next generation emissions measurement (NGEM) and modeling approaches that can help identify and reduce emissions, advance knowledge of impacts, and improve transparency for near-source communities [7,8].

Encompassing a wide range of techniques, some NGEM tools are deployed in shorter-term intensive campaigns to quantify emission rates and assess spatial impacts [9,10], while others can be used more continuously to monitor for the onset of abnormal emission events [11,12]. NGEM can be executed in facilities to support enhanced emission detection and repair strategies and at the fence line and beyond to assess different aspects of emissions and effects [13,14]. Enabled by the emergence of lower cost sensors, instruments and mobile technologies, new generators of SIS-relevant NGEM data can be envisioned [15]. Explored here is the complementary use of NGEM systems possessing a range of temporal resolution, speciation capabilities, and implementation costs that can help inform SIS emissions from remote vantage points in long-term observation.

In this project, the United States Environmental Protection Agency’s (EPA’s) Office of Research and Development (ORD), EPA Region 4, and the City of Louisville Metro Air Pollution Control District (LMAPCD), are working together to demonstrate emerging NGEM approaches in the Rubbertown industrial area of west Louisville, KY. The area has faced challenges related to the control of ozone, potential exposure to hazardous air pollutants (HAPs), and reoccurring odor issues. Rubbertown has a concentration of chemical facilities, commercial and industrial operations, and a municipal waste water facility servicing a combined sewer and water system. The area also includes a Superfund site and numerous brownfield sites where past waste disposal has occurred. Spatial clusters of elevated asthma rates in Louisville have been documented [16,17], and relatively high concentrations of certain HAPs in Rubbertown neighborhoods were observed in the West Louisville Air Toxics Study (WLATS), conducted from 2001 through 2005 [18]. In direct response to the latter, LMAPCD adopted the Strategic Toxic Air Reduction (STAR) Program in 2005, which required Rubbertown facilities emitting certain HAPs to demonstrate that their emissions are environmentally acceptable using screening methods and EPA-approved modeling techniques [19,20]. Although emissions of many HAPs have been reduced as a result of the STAR Program, air toxics such as 1,3-butadiene remain a source of concern. Periodic significant odor episodes exhibiting both “waste” and “chemical” characteristics are an ongoing issue and serve to increase community concerns about exposure to air pollutants.

With field deployments beginning in September 2017, the Rubbertown NGEM Demonstration Project is a multi-year (at limited scope) effort that seeks to develop and test emerging NGEM systems and inform aspects of Rubbertown SISs to the extent possible. Due to the prototype nature of the methods employed, the project does not attempt to identify or statistically test any formal air quality, public health, or source-impact hypothesis.

A primary goal of the project is to explore how NGEM approaches with different measurement performance capabilities can work together to inform aspects of source emissions and effects. This effort focuses on select volatile organic compounds (VOCs) and HAPs and consists of four main components: Geospatial deployment of time-integrated passive samplers (PSs), investigation of fenceline sensors (FSs) with canister sampling, testing new fieldable gas chromatographs (GCs), and the exploration of source attribution approaches.

This paper provides illustrative examples of emerging NGEM technologies. The sampling locations, measurement technologies and methods used in the first year of the Rubbertown NGEM Demonstration Project are described. With a focus on 1,3-butadiene, long term PS data are reviewed and two case study examples of SIS emission events that affected the local air shed to different degrees are analyzed. The collaborative value of multiple types of NGEM data and source location models to improve source understanding and exposure information is discussed.

## 2. Methods

### 2.1. Sampling Locations

**Figure 1:**
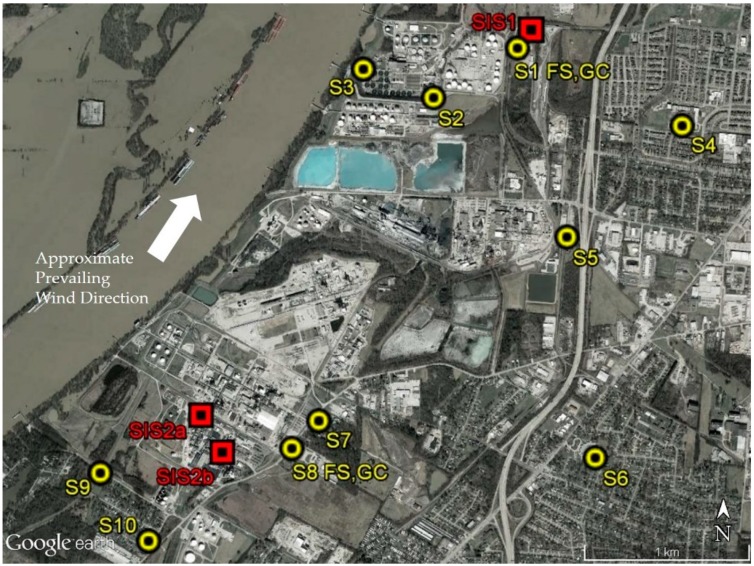
Measurement sites for the first year of operation of the Rubbertown NGEM Demonstration Project. Yellow circles indicate primary measurement sites. Red squares indicate approximate locations of the example short-term SIS emissions events.

**Figure 2:**
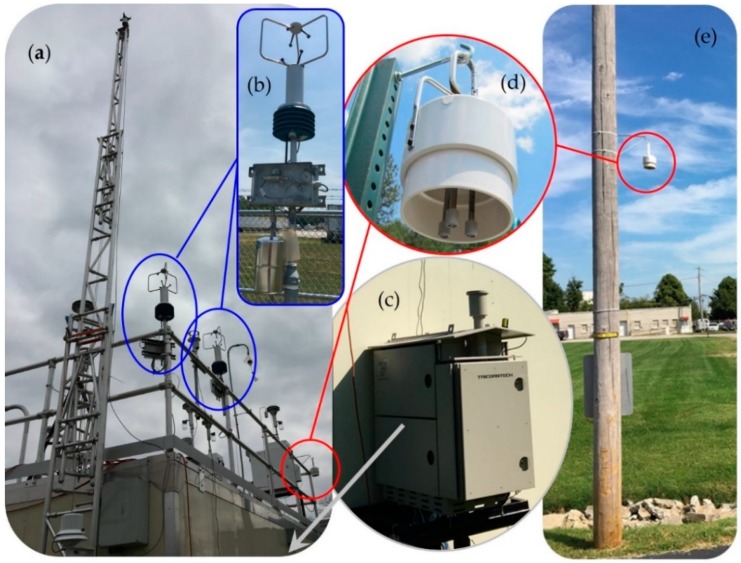
NGEM systems located at (a) S1, LMAPCD monitoring shelter housing auto-GC and other instruments, (b) one of two EPA SPod FSs with ACGS, (c) MiTAP field GC (off frame low), (d) PS at S1 (left circle) and close-up of the PS deployment system (center circle) and, (e) PS at S10 illustrating an elevated deployment in a public area (right circle).

Primary measurement sites (S), labeled S1–S10, are shown in Figure 1 with detailed information provided in Supplementary Materials (SM), Table SM1. Additional information on major Rubbertown facilities is available elsewhere [21]. Passive samplers were deployed continuously at S1–S10 from 12 September 2017 (9/12/2017) to 9/12/2018. Each PS deployment cycle was nominally 14 days in duration, yielding 26 total sampling periods. One PS site, S11 (not shown in Figure 1), was added to the north of the primary project area from 6/19/2018 to 9/12/2018 (6 PS periods). All PSs were located close to a facility fenceline (<0.5 km), except S4, S6, and S11 that were deployed at neighborhood schools >1 km from the nearest Rubbertown facility. Prototype FSs and field GCs were deployed at S1 and S8 for part of the first year. S1 is the location of the permanent LMAPCD Firearms Training Center (FTC) monitoring site and contained the LMAPCD auto-GC, a field GC (MiTAP), and FSs (Figure 2). Time-resolved FSs began operation at S1 on 9/13/2017 and at S8 on 6/4/2018 with a field GC moved from S1 to S8 and made operational on 7/25/2018. A limited number of manually activated and automated canister grab samples, (MCGS and ACGS respectively), were acquired in the first year of the project with the ACGS executed only at S1 and S8, using acquisition triggers provided by FSs and a field GC. Wind roses from local LMAPCD monitoring sites are contained in Supplementary Figure SM1, with the approximate dominant wind direction indicated in Figure 1.

### 2.2. Passive Samplers (PSs)

One of the simplest and lowest cost NGEM approaches involves geospatial deployment of time-integrated PSs around potential emission sources (Figure 2d,e). PS deployment and analysis methods for the compound benzene are well-developed and are part of a fenceline monitoring work practice currently being implemented by U.S. refineries [14,22]. The use of PSs around sources of other air pollutants, such as 1,3-butadiene, is less well established and one objective of this project is to advance these methods. The diffusive tube PS sampling media, preparation, shipping, and deployment procedures used here were similar to that employed in previous efforts [23,24,25,26]. In brief, the PSs were 89 mm long, 6.4 mm outer diameter passivated stainless steel tubes, (part number (PN) 28686-U, Supelco FLM Carbopack X deactivated TD tube for fenceline monitoring, Sigma-Aldrich, St. Louis, MO, USA), with 6.4 mm brass Swagelok® endcaps, one-piece Teflon ferrules, and diffusion caps (PN L4070207, PerkinElmer, Shelton, CT, USA). Prior to deployment, the PSs were conditioned in the laboratory (75 mL/min helium purge at 350 °C for 1 hour) and shipped to LMAPCD in a cooler (no ice) to provide some thermal stability and physical protection. In the field, the brass endcap on the sampling end of a PS was removed and replaced with a diffusion cap to expose the sorbent to ambient air. The PSs were mounted in a protective housing using a snap-in aluminum holder (Figure 2d), then the housing was secured to a structure at the site with an elevated deployment strategy used for S4, S6, S10, and S11 (e.g., Figure 2e, Table SM1). After nominally 14 days of air exposure, the PSs were capped and shipped to the EPA ORD’s laboratory for analysis by thermal desorption/GC time-of-flight (TOF) mass spectrometry using a Markes International Inc. (Gold River, CA, USA) ULTRA 2 autosampler and UNITY 2 thermal desorber interfaced to an Agilent 7890B gas chromatograph/Markes Bench TOF-Select mass spectrometer. The field change-outs of the PSs required three hours and were typically executed between 9:00 and 14:00. When a set of PSs was retrieved, a new unexposed set was deployed. A total of 26 compounds was quantified in the PS analysis with this paper focusing on 1,3-butadiene. The average method detection limit (MDL) for 1,3-butadiene for this project was 0.010 ppbv. The PS portion of the project used duplicates, field blanks, and field spikes as primary quality control checks, and further information on the method is contained in text associated with Figure SM2. The deployment of two-week PSs for 1,3-butadiene measurements in communities is considered a test application of the fenceline EPA Method 325 [14] approach and all factors affecting accuracy are not known. Previous research has shown that uptake rates for 1,3-butadiene decreased by approximately 18% for controlled 7-day versus 14-day exposure chamber studies [27].

### 2.3. Fenceline Sensors (FSs)

Use of time-resolved, non-speciating photoionization detector (PID) sensors deployed at fencelines is an emerging NGEM approach for the detection and location of VOC and HAP sources [25]. The primary FSs used in the first year of this project were EPA SPods, (Figure 2b). The SPod combines pollutant concentration information from a high sensitivity 10.6 eV PID (PN 045-014, Baseline-Mocon, Inc., Lyons, CO, USA, or PN MiniPID2-HS, Ion Science Inc., Stafford, TX, USA) with time-synchronized wind data measured by an ultrasonic anemometer (PN 81000V, R.M. Young, Inc., Traverse City, MI, USA), to allow the magnitude and direction of the origin of the air pollutant source to be assessed. The SPod PIDs produce an uncalibrated VOC plume detection signal in mV, digitized to 16 bits, and reported as “counts” (cts). The SPod signal corresponds to the integrated mixing ratio of the ionizable species in the detected plume (typically possessing a range of PID response factors). To provide a sense of SPod sensitivity, a 500 ppbv isobutylene spike test produces ~2000 cts to 4000 cts (~1/20 scale), above baseline, with typical MDLs ranging from ~ 0 cts to 150 cts, depending on the type of PID employed, its age, and ambient conditions.

The SPod uses an EPA-developed sensor board that supports the PID and a temperature, relative humidity and atmospheric pressure sensor package (PN 2652, Adafruit Industries LLC, NYC, NY, USA). The SPod is controlled by a low cost, low power consumption Arduino® data logger (PN Teensy 3.2, Adafruit Industries) that records the 1 Hz combined data stream to an onboard Secure Digital (SD) memory card. SPod nodes typically communicate using a short-range ZigBee® (IEEE 802.15.4) network with a weather-proof base station module fitted with a microcomputer/gateway (PN V2201, Moxa Americas Inc, Brea, CA, USA) running custom data acquisition software (EPA VOC Emission Tracker v1.0.4, LabVIEW National Instruments Inc, Austin, TX, USA) and cell phone modem (PN MC7354, Sierra Wireless, Richmond, British Columbia, Canada) to externally communicate data. The baseline stability of SPods used in this project were improved over previous versions by reducing humidity effects on the PID through the incorporation of a polyimide strip heater (PN HK6903, Minco Minneapolis, MN, USA) running at ~30 °C. Starting in April 2018 the SPods were fitted with ACGS trigger systems allowing ~35 second duration samples to be acquired in cleaned and evacuated 1.4-liter Silonite® canisters (PN 29-MC1400SQT, Entech Instruments, Simi Valley, CA; Figure 2b). Analysis of canister samples was by EPA TO-15 [28], with the range of locations, times, and general findings described in a future publication. The EPA open-source SPod design, software, and use information is available upon request to the corresponding author.

### 2.4. Field Gas Chromatographs (GCs)

Emerging Field GCs for NGEM applications range from fence-mounted low-cost units (< $25,000 USD) with single source detection goals, to self-contained mid-cost units (< $100,000 USD) possessing improved detection limit and speciation capabilities, to higher performance systems housed in traditional walk-in air monitoring shelters. The first year of this project successfully deployed a mid-cost field GC (MiTAP^®^ P310, Tricorntech Corp. Taipei City, Taiwan; Figure 2c) and an early configuration of the auto-GC system, customized for Rubbertown-specific compounds (Airmo VOC C3-C6, AirmoVOC C6-C12, Chromatotec America Inc., Houston, TX, USA; shelter-integration by Consolidated Analytical Systems, Cleves, OH, USA; Figure 2a), deployed by LMAPCD. The deployment of a prototype low-cost benzene GC was also briefly attempted but produced no usable data.

The small form factor MiTAP GC was housed in a weather-proof enclosure (Figure 2c), containing carrier and calibration gas, an automatic calibration check system, and an ACGS trigger system allowing ~one-minute duration samples to be acquired in a 1.4-liter Silonite^®^ canister via GC trigger. The MiTAP sampled air for 15 minutes of each hour, producing 22 hourly values per day, with the calibration check taking two hours per day. The MiTAP GC was configured to quantify 12 compounds, (selected 1,3-butadiene results discussed here), with five other compounds tracked as near coeluting interferents. The on-board calibration cylinders of the MiTAP (five in total) contained between 4.6 ppbv and 8.6 ppbv 1,3-butadiene, as determined by lab testing. A conservative manufacturer-set MDL was 0.1 ppbv for this compound (the reporting limit). The MiTAP GC and calibration cylinder concentrations were tested at the EPA VOC laboratory and were found to have a low bias; however, due to the extended range of data presented here (well above calibration range with uncertainties in linearity), no additional bias correction factor has been applied.

In its initial deployment, the LMAPCD auto-GC system was set up to produce two data points per hour with air sampled for 22.5 minutes of each 30-minute period. The GC used for 1,3-butadiene measurements employed permeation tube checks with n-butane, n-hexane, and benzene sequentially twice per day to measure retention times. Accuracy comparisons to EPA-prepared standards in the 5 to 10 ppbv range were executed on several occasions. The LMAPCD GCs have the potential to automatically quantify dozens of VOC and HAP compounds relevant to Rubbertown, but the initial deployment of the system found significant issues with stable operation caused by factors including, but not limited to, humidity-related retention time shifting and difficulties with coeluting compounds exacerbated by proximity to a fuel storage terminal and waste water treatment facility.

The chromatography of 1,3-butadiene was sufficiently separated from interfering species, so it could be positively identified and integrated with confidence. The 1,3-butadiene MDL for this analysis was estimated at 0.1 ppbv. Due to the prototype nature of both GC systems, data availability for the first year of deployment required significant post processing and expert analysis.

### 2.5. Source Location Models

An important aspect of this project is to explore how NGEM data can be used with geospatial analysis and source location models to inform emissions and impacts. This paper describes several preliminary approaches through the discussion of two example case study SIS events. The simplest form of emission source visualization used was angle-resolved display of the time-synchronized concentration and wind data from the SPod FSs. Called a source direction indicator (SDI) plot, this time-integrated approach provided first-level source direction information with low computational burden. Emerging back trajectory models (BTMs) use wind field information and atmospheric data to simulate the spatially-resolved transport of emissions in a kilometer-scale domain. In this paper a prototype Lagrangian/CALMET BTM, produced by EnviroSuite LLC (Brisbane, QLD, Australia) was used to help understand the example SIS events. The three-dimensional CALMET wind field was set at 150 m grid spacing and was operated at five-minute time resolution using one-minute average wind data. The BTMs were additionally informed using a new approach for identifying the origin of emissions called temporally combined trajectory analysis (TCTA). For the example SIS events, a trial TCTA source location was selected, and the number of discrete BTM points that intersected a defined source area was determined and compared against measured concentrations over time. The defined source areas were 10 m or 20 m radius circles for cases where the trial source location was <200 m from the observation point and 50 m in radius otherwise. The SDI, BTM, and TCTA approaches did not consider wind flow effects around obstructions near the NGEM monitoring locations. The dynamics of emission plume pooling and flow around buildings and structures for the case studies were investigated using the Los Alamos Quick Urban & Industrial Complex (QUIC) Dispersion Modeling System. Further descriptions of these approaches are provided at the point of reference with details in the Supplementary Materials.

## 3. Results and Discussion

### 3.1. Passive Sampler Measurements

**Figure 3:**
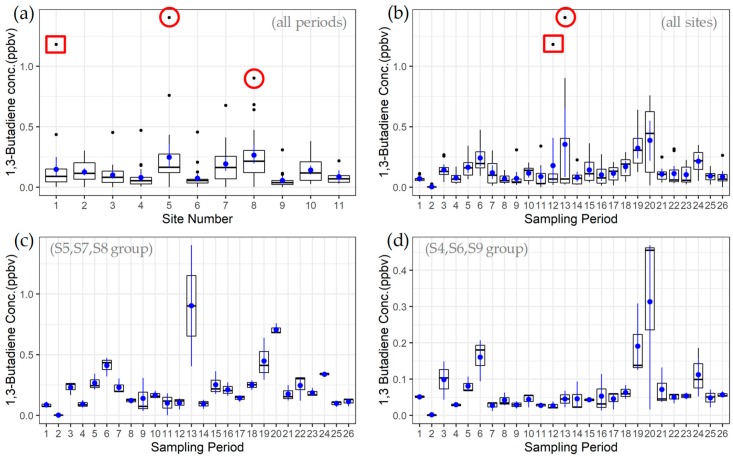
PS-determined 1,3-butadiene concentrations: (**a**) By monitoring site with all 26 PS sampling periods grouped [6 periods for S11], (**b**) by PS sampling period with all 10 monitoring sites grouped, (**c**) by period with three sites closest to 1-3 butadiene-relevant facilities grouped (near-facility group), and (**d**) by period with two farthest sites and an upwind site grouped (community/upwind group). The box whiskers extend to the largest measurement <1.5 times the interquartile range. Blue markers indicate means with 95% confidence intervals calculated by non-parametric bootstrap. The red box and circles in (**a**) and (**b**) indicate the highest reading at S1 during period 12 and highest readings at S5 and S8 during period 13, respectively.

Exhibiting both temporal and spatial variability, the two-week average 1,3-butadiene PS concentration data for the 26 sampling periods of the first year of the project are summarized in Figure 3 and Table SM2. The three measurement sites located closest to facilities that emit 1,3-butadiene (S5, S7, and S8) exhibited the highest average PS concentrations (Figure 3a,c, and Figure SM3). These three sites, called the near-facility group, possessed a grouped mean (and standard deviation [σ]) of 0.24 [0.20] ppbv. The near-facility group produced the highest PS reading for 70% of the PS sampling periods, with S10 and S2 accounting for 25% of the highest period readings. With expected lower concentrations, the sites that were farther removed (S4 and S6) or generally upwind (S9) of facilities were grouped together and designated as the community/upwind group (Figure 3d). S11 was also located away from facilities, but was only deployed for six periods, so it was not considered part of the community/upwind group in this analysis. With a grouped mean [σ] of 0.07 [0.06] ppbv, the community/upwind group produced the lowest concentrations in 90% of the PS periods. Using a Wilcoxon-Mann-Whitney test, the differences in medians for the near-facility and community/upwind groups was statistically significant at the 99% confidence level (*p* < 0.01).

The near-facility group was noticeably elevated during PS period 13 (3/2/2018 to 3/14/2018), producing the highest (1.40 ppbv) and third highest (0.90 ppbv) PS observations for the project at S5 and S8, respectively (red circles, Figure 3a,b). The third member of the near-facility group (S7) was also elevated at 0.40 ppbv during this period. However, other PS sites, including the community/upwind group, were not strongly elevated during period 13, indicating a relatively localized effect in this case. At S1, for example, the period 13 PS and the period time averaged LMAPCD GC and MiTAP GC readings for 1,3-butadiene were 0.04 ppbv, 0.03 ppbv, and <0.01 ppbv, respectively. For this comparison, the GC values below MDL were set to 0 ppbv and the MiTAP values carry a QA flag since the daily calibration was not operating during this period.

Spatially localized 1,3-butadiene concentrations were also observed during PS period 12 (2/13/2018 to 3/2/2018), where the second highest PS observation for the project (1.18 ppbv) was recorded at the FTC monitoring site (S1) and is ascribed to example emission event SIS1. At over seven times the S1 PS average, this observation represents a clear outlier for that site (red box, Figure 3a). This elevated concentration was also spatially distinct (red box, Figure 3b), as evidenced by comparing the 1.18 ppbv result to the mean [σ] of all other PS locations for that sampling period, (0.07 [0.04] excluding S1). This separation from other PS data is indicative of either a temporary source proximate to S1, or a significant PS method issue for that observation, with the latter concern partially alleviated through comparisons to other NGEM equipment. For example, the LMAPCD and MiTAP GCs at S1 also registered their highest average 1,3-butadiene concentrations during period 12 at 3.3 ppbv and 2.6 ppbv, respectively with the results dominated by a few strongly elevated readings that exceeded 100 ppbv.

The difference in absolute levels between the GCs and PS in this case may be due to several factors including sampling time differences between the GCs, potential linearity, calibration uncertainty, and residual compound carry-over issues with the GCs due to the abnormally high concentrations encountered, and proximate source plume coupling differences between the GCs and the PS. The PS period 12 deployment occurred during heavy sustained rains that resulted in area flooding. At 17 days, the PS deployment was longer than typical, and the abnormally wet conditions may have impacted the measured values of 1,3-butadiene.

The largest spatial extent of elevated 1,3-butadiene concentrations was observed during PS period 20 (6/6/2018 to 6/19/2018) and was likely influenced by a known emission event at a facility west of S8 in the area near SIS2a and SIS2b of Figure 1. Time-resolved aspects of this event are discussed as case study SIS2 in Section 3.3. For period 20, the near-facility PS group was uniformly elevated with a mean [σ] of 0.71 [0.05] ppbv. With winds primarily from the west/southwest and southwest during period 20 (Figure SI1d), S1, S4 and S6 were downwind from the emitting facility, and exhibited 1,3-butadiene concentrations of 0.44 ppbv, 0.47 ppbv and 0.46 ppbv, respectively. These results were significantly higher than their individual site averages, and together with the near-facility group, provide a sense of the spatial extent of the elevated concentrations observed during this period. During period 20, S9 was located upwind of this facility and registered a low result of 0.02 ppbv 1,3-butadiene, producing a somewhat clear indication of upwind versus downwind effects. The S1 PS period 20 1,3-butadiene result of 0.44 ppbv agreed favorably with the average over the same period of the LMAPCD GC (0.49 ppbv) and the MiTAP (0.43 ppbv). In this case, the MiTAP GC was offline for the first five days of the PS period 20 and data from LMAPCD GC was used to fill the missing values for the estimation of the MiTAP average.

The PS results from this project can be compared to the WLATS [18] that acquired 24-hour canister measurements on an approximate 1 in 12 day schedule with study sites A and F, being similar locations to S1 (FTC) and S6 (Cane Run Elementary school) used in this study. Table 1 expresses approximate WLATS results in ppbv at standard conditions (μg/m^3^ to ppbv at 25 °C, 1013.25 mbar). Since the PS approach continuously samples ambient air, the techniques differ greatly in temporal coverage with the WLATS representing on average <9% of sampling days per year. Because the PS data represent a two-week time average, maximum PS values are expected to be lower than the maximum 24-hour canisters acquired in source-impacted areas. With these differences in mind, it is evident that NGEM PS results are markedly lower than those observed in WLATS. This may be due to reductions in both stationary source and mobile source contributions to the airshed that occurred in the intervening decade, with the latter due to fleet trends. The maximum PS reading at S1 occurred during SIS1, and with this reading excluded, the second highest reading at S1 occurred in period 20, during SIS2. The maximum PS reading at S6 also occurred during period 20. Due to 1,3-butadiene PS method factors (Figure SM2), absolute comparisons to low-level benchmark ambient concentrations (BACs) carries significant uncertainty.

### 3.2. SIS1 Event

**Figure 4:**
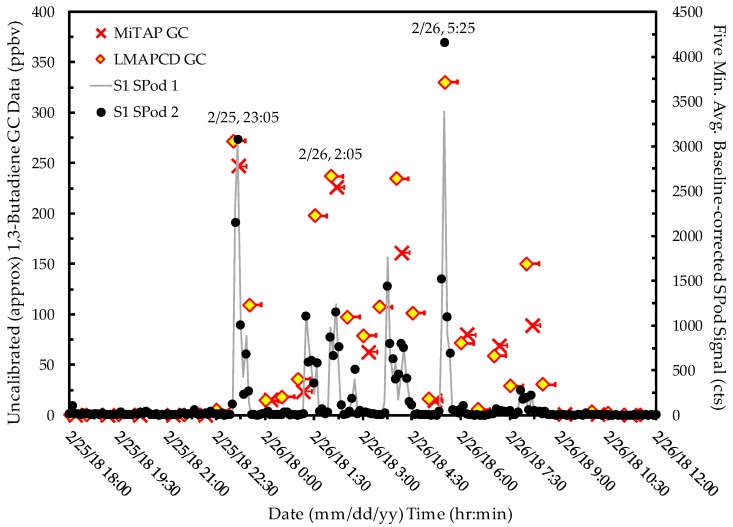
Time-resolved measurements of SIS1 event with SPod data averaged to five minutes. Single side horizontal error bars in GC data indicate the temporal extent of air sampling. Due to observed concentrations well beyond instrument calibration ranges, the GC data are considered approximate.

As a first case study of the complementary use of multiple NGEM systems, time-resolved pollutant concentration and wind measurements at S1 can help inform the elevated PS period 12 reading and provide some indication as to source origin. The SIS1 event was characterized by a series of short time duration, strongly elevated 1,3-butadiene concentration episodes that were simultaneously observed by the LMAPCD GC, the MiTAP GC, and two SPod FSs. An elevated 1,3-butadiene signal from the SIS1 event was briefly observed during the late afternoon on 2/24/2018 under modest wind speeds as heavy area rains subsided. This discussion focuses on the time period where the majority of SIS signal was observed, from 23:00 on 2/25/2018 to 8:00 on 2/26/2018 (Figure 4). With some values approaching ~330 ppbv, these 1,3-butadiene readings were by far the highest observed by either GC or canister grab measurement for the project. These values were well above the normal calibration and linearity range of the GCs so they are approximate. The two GCs recorded similar values when differences in air sampling frequency and duration are considered. The two independent SPod FS readings were also similar to GC readings for many of the episodes with departures due in part to sample integration time differences and SPod baseline correction algorithm settings that artificially depress background VOC levels (slow signal) in order to enhance the near-field emission plume detection capability (fast signal).

No MCGS or ACGS was available for this event, but the GC data did not indicate the presence of co-emitted compounds associated with the SIS1 event. These significantly elevated readings were in part due to extremely low wind speeds (<0.5 m/s) at the time of measurement (Figures SM4 and SM5), along with typically compressed boundary layer conditions in nighttime hours. Based on wind information and the modulated temporal profile of the measured concentrations, a proximate 1,3-butadiene source emitting into a stagnant air mass with periodic puff-flow to S1 is suggested. This assessment agrees with the isolated nature of the period 12 S1 PS result discussed in Section 3.1.

**Figure 5:**
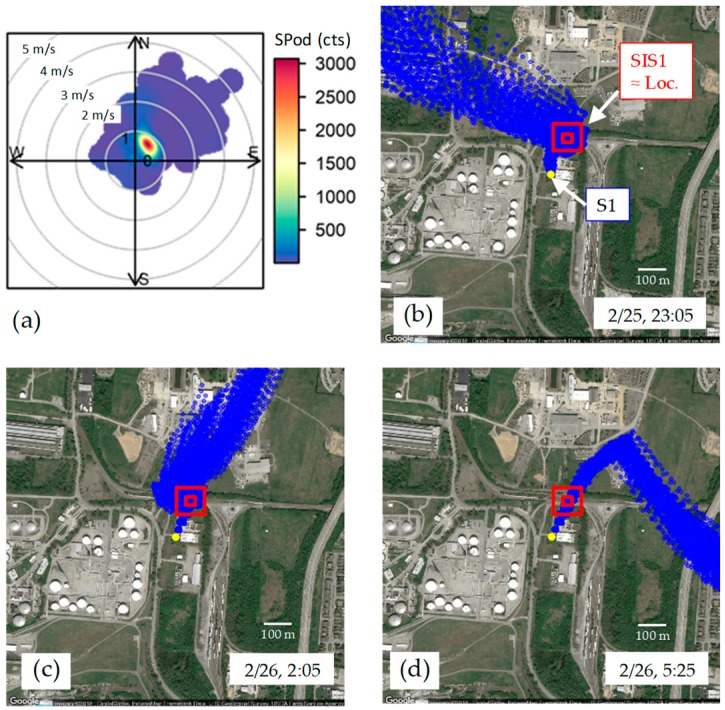
(**a**) S1 SPod source direction indicator (SDI) plot for 22:00 on 2/25/2018 to 11:00 on 2/26/2018 with concentric circles representing wind speed in m/s and color bar SPod PID, and (**b**), (**c**), and (**d**) showing 99 back trajectory paths each for the five-minute BTM runs for 23:05 on 2/25/2018, 2:05 on 2/26/2018, and 5:25 on 2/26/2018, respectively, the highest concentrations of Figure 4. The concentric red boxes represent the near-optimal trial source location from TCTA with the inner box approximating the defined source areas for the TCTA calculation.

Investigating the origin of SIS1 emissions, Figure 5a shows an SPod SDI plot for the event indicating the presence of a VOC emission source located to the northeast of S1 (not orginating from the facilities to the south). Figure 5 also shows three prototype EnviroSuite BTMs (approach further detailed in Figure SM6) for times near the three highest GC readings of Figure 4 (23:05 on 2/25/2018, and 2:05 and 5:25 on 2/26/2018). A potential source location for SIS1 (red double square in Figure 5b–d) is indicated and was determined by comparing similarities in multiple BTM paths with the occurrence of elevated concentrations (a precursor to TCTA). The “bunching” of trajectories just north of S1 in Figure 5b is indicative of a lull in wind speed (Figure SM5) where emissions likely pooled just prior to transport to S1, resulting in large measured concentrations. Under these challenging low wind speed conditions, some of the BTM runs exhibit nonphysical properties (e.g., sharp transition in Figure 5d); however, the proximity of the source (likely within 200 m of S1) limits the impact of plume transport uncertainty in this case.

As discussed in the Supplementary Materials, the TCTA is a prototype approach for identifying the origin of detected SIS emissions by comparing the spatial overlap of a potential source location with a larger series of BTM runs and time-resolved NGEM data. An example of TCTA for SIS1 for the subject time periods is contained in Figure SM7 and Supplementary Video 1 (Video SM1). The TCTA approach is rudimentary at present and will be further developed with data from this project. As an example of one technical issue with TCTA, the current BTMs do not account for wind flow around obstructions, such as the buildings and structures to the north and northeast of S1 (Figure SM8). These structures effectively channel flow from a proximate source limiting TCTA source location certainty. The dynamics of emission pooling and flow of the plume around structures near S1 for this event were investigated in a preliminary fashion using the QUIC model (Video SM2).

Although it is clear that SIS1 did not originate from the facilities to the south of S1, the identity of SIS1 remains unknown. There are no 1,3-butadiene emitting facilities located to the north of S1. As shown in Figure SM8, railcars that may transport 1,3-butadiene can park for extended periods in the general area where the emission is thought to have originated, and cameras have been installed at S1 to observe the areas to the north of the site. The 2/24/2018 to 2/26/2018 SIS1 event coincided with significant area flooding. On 5/7/18, after very heavy rain, a brief occurrence of elevated 1,3-butadiene concentrations was observed from the same SIS1 origin direction (Figures SM9 and SM10, and Video SM3); however, this event did not significantly impact the associated S1 PS reading. Subsequent to the second observation, PS S11 was installed to the north of S1 to provide additional diagnostic power. Elevated 1,3-butadiene was not observed after a heavy August 2018 rain event with winds from the north (with a lower water table). Several courses of investigation are underway and the ability to continually monitor for SIS1 illustrates a strength of long-term NGEM approaches over temporally intensive studies.

### 3.3. SIS2 Event

**Figure 6:**
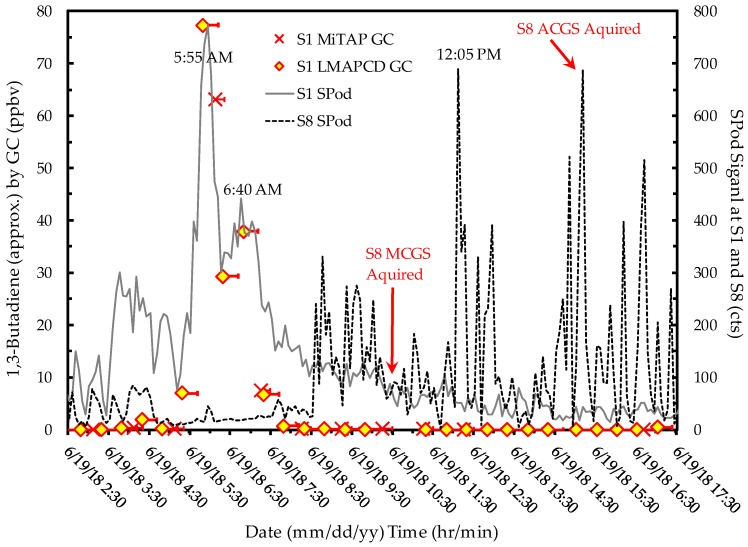
Time-resolved observations of SIS2 with SPod readings (five-minute average) plotted on the secondary ordinate axis. Single side horizontal error bars in GC data indicate the temporal extent of air sampling. The SPod data are non-speciated, and do not directly correspond to 1,3-butadiene.

A second example case study of the utility of multiple NGEM approaches is the assessment of an SIS event at a Rubbertown facility that likely contributed to the elevated 1,3-butadiene observations of PS period 20 (Section 3.1). Figure 6 presents S1 SPod, S1 GC, and S8 SPod data from 6/19/2018. At ~75 ppbv, the second highest 1,3-butadiene concentration (aside from the SIS1 events) for the first year of the project was observed around 6:00 on 6/19/2018, with the GC data being similar between the two units when sampling time differences are considered. The overnight compressed boundary layer and low wind speeds (~1 m/s from the south/southwest; Figure SM11) contributed to the elevated readings at S1. The S1 SPod data of Figure 6 are presented with no baseline correction to illustrate the broadly elevated VOC levels observed at S1 (downwind from Rubbertown facilities) in the early morning compared to the S8 SPod sampling relatively source-free air prior to 8:00 on 6/19/2018 (Figure SM6). As discussed subsequently, the general decrease in signal at S1 with corresponding increase in signal at S8 coincides with a mean wind direction change that allowed SIS2 to be observed from multiple vantage points.

**Figure 7:**
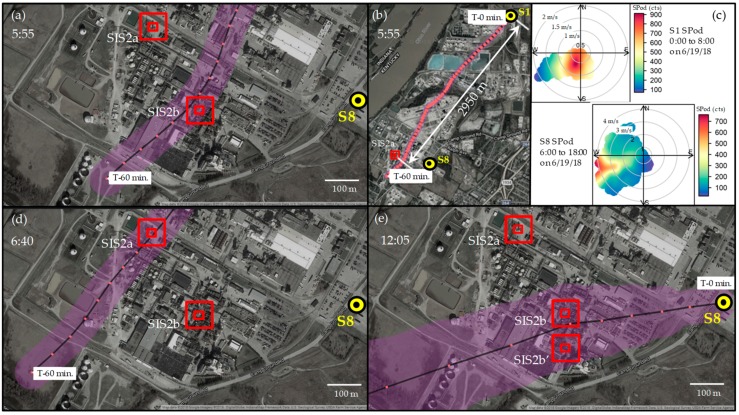
BTMs of SIS2 on 6/19/2018: (**a**) expanded view of 5:55 model from S1 (**b**) full view of 5:55 model from S1, (**c**) SPod SDI plot from S1 (top) and S8 (bottom), (**d**) expanded view of 6:40 model from S1, and (**e**) expanded view of 12:05 model from S8 with QUIC model optimized location SIS2b’ indicated. The concentric red boxes represent the near-optimal trial source location from TCTA, with the inner box approximating the defined source areas for the TCTA calculation.

The SIS2 event illustrates the importance of speciated NGEM data acquired at both S1 and S8. In this case, confirmation of the identity of the facility emitting the 1,3-butadiene was provided by the LMAPCD GC at S1 through observation of a temporally-correlated cyclohexane signal at similar concentration levels. Based on this information, LMAPCD contacted the only facility in Rubbertown that could co-emit this combination of compounds, and the facility confirmed the occurrence of an SIS condition involving an emissions control system that required a change in operation. The position of the control device is listed as SIS2a in Figure 1 and Figure 7, whereas a general location of the process unit for the facility is shown as SIS2b. As indicated in Figure 6, at S8 a MCGS was acquired at 10:21 and a SPod acquired an ACGS at 15:12 during a large spike in VOC concentration. The MCGS, acquired at lower overall VOC levels, indicated 1.4 ppbv and 7.6 ppbv concentrations of 1,3-butadiene and cyclohexane, respectively. The ACGS produced instantaneous concentrations of 12.5 ppbv 1,3-butadiene and ~400 ppbv cyclohexane, respectively, confirming that at least part of the S8 SPod signal can be ascribed to SIS2.

Figure 7 shows a combination of SPod SDI plots and BTMs for the SIS2 event. The VOC source(s) to the south of S1 are represented in the early morning SPod SDI plot of Figure 7c (top panel). Around 8:30, the wind direction changed to westerly, and the wind speed increased to ~ 2 m/s (Figure SM11). As indicated in Figure 6, this change coincided with a decrease in signal at S1 and a sharp rise in S8 SPod signal, as that monitoring site was then approximately downwind of the SIS2 source(s) (Figure 7c, bottom panel).

Figure 7b shows the full view of a BTM originating at S1 for 5:55 on 6/19/2018, near the time of maximum concentration observed at S1. This model calculation is the same as that presented in Figure 5, except only the mean trajectory (average of 99 individual paths) is displayed, with the red data points showing the one-minute progressive time steps, and the approximate uncertainty band indicated by shading. As described in Figure SM6, the uncertainty in mean trajectory is believed to be underestimated for some conditions. Figure 7a is an expanded view of Figure 7b showing the end of the trajectory model passing near the main processing unit of the facility, generally indicated as SIS2b. The selected location for SIS2b along the direction of plume propagation is somewhat arbitrary and was chosen here to be towards the north end of the process unit for TCTA comparisons because the 60-minute modeled period for the BTMs projected from S1 barely reach the facility. This is evident in the initial BTMs of the multi-hour time series provided in Video SM4.

As described in the Supplementary Materials (Figures SM6 and SM12), the prototype TCTA analysis for SIS2 indicates that the high concentration readings observed at S1 from 5:00 to 6:00 on 6/19/2018 may correspond to a set of trajectories passing near location SIS2b (e.g., Figure 7a), whereas the elevated readings around 6:40 may be a better match for trajectories passing near potential source location SIS2a (e.g., Figure 7d). The ability of a 3 km distance BTM to decipher the spatial differences between SIS2a and SIS2b is unproven at present and would likely only be possible under the most favorable atmospheric conditions and detection scenarios. Based on current information we cannot say with certainty that the long range BTMs from S1 indicate two emission source locations within the facility. As the day progressed, the trend to unstable atmospheric conditions greatly increased BTM uncertainty (Figure SM6, Video SM4), making precise multi-kilometer BTMs and robust TCTA analysis impossible.

From a closer vantage point at S8, the SPod SDI plot (Figure 7c, bottom panel) significantly departed from the SIS2a position and indicated that a source south of SIS2a was the major contributor to the non-speciated SPod signal. Figure 7e shows a higher uncertainty S8 BTM corresponding to the 12:05 elevated SPod reading (Figure 6) with a mean trajectory passing just south of the initially-selected SIS2b location. With complementary uses of NGEM tools, some optimization of the position of SIS2b along the direction of plume propagation can be attempted and is represented here as SIS2b’, 100 m south of SIS2b, Figure 7e.

**Figure 8:**
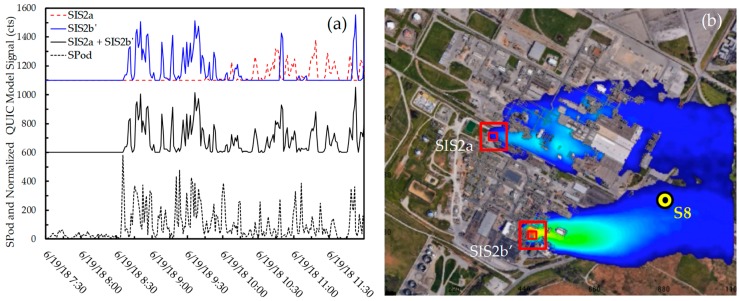
(**a**) Comparison of one-minute time average SPod readings from S8 (bottom panel) with QUIC model simulated emission time series from SIS2a (red dashed trace top panel), SIS2b’ (blue trace top panel), and a composite result of SIS2a and SIS2b’ (middle panel), (**b**) frame of QUIC model video (Video SM5) at 10:15 on 6/19/2018.

To check the relative contributions of SIS2a and processing unit source (SIS2b or SIS2b’) and to inform the location of the latter, dual-source QUIC model runs were employed (Video SM5). The SIS2a and the optimized SIS2b’source locations were found to compare favorably to the SPod-measured time series (Figure 8). Here the QUIC results were scaled to the SPod and the panels were offset for ease of viewing. For simplicity, the two sources were assumed to emit at the same constant rate with SIS2a modeled as a 30 m elevated stack release and SIS2b’ as a ground source. Figure 8a shows both the individual QUIC-modeled results for SIS2a and SIS2b’ (top panel) and the composite result (middle panel). The QUIC model result indicated a better overall match for the SIS2b’ location, compared to the original SIS2b location (Figure SM13). The difference in the SIS2a and SIS2b’ contribution to the overall signal is similar in form to that represented by the SDI plot. Although the QUIC model accounts for wind flow obstructions at some level, the result has not been corrected for variable plume transit time from the source locations to S8, which would tend to improve temporal matching.

Using both speciation and inverse source location techniques, NGEM approaches at S1 and S8 detected emissions on 6/19/2018 that likely originated from a 1,3-butadiene-emitting facility that experienced an acknowledged temporary emission control issue. TCTA analysis of BTMs from S1 suggest multiple source locations in the facility may be possible and may include the potential location of the control issue (SIS2a). The S8 SPod signal registered concentrations from the process unit area near SIS2b (more optimally SIS2b’), but the degree to which these emissions were transported to S1 are uncertain. Although the precise assignment of two specific SIS locations within the facility cannot be made with certainty in the current analysis, the case study is valuable for continued development work. From this example, the potential power of multiple speciated NGEM measurements and models is illustrated. It is also clear that the addition of spatially separated NGEM sensor nodes would significantly increase inverse source triangulation diagnostic power.

## 4. Conclusions

This paper provided illustrative examples of new measurement technologies and source location models that are being explored in the Rubbertown NGEM Demonstration Project. A primary objective of the effort is to learn how emerging NGEM systems may ultimately work together to provide enhanced information to regulators, industry and communities. An overview of NGEM techniques used in the first year of the project was discussed with a focus on 1,3-butadiene, a HAP emitted in Rubbertown. Two case study examples of emission events illustrated how time-integrated (PSs) and time-resolved (FS, GC) NGEM approaches, along with source location models, can provide complementary assessments to improve SIS understanding.

One year of PS deployments at 11 locations around Rubbertown provided useful data on the levels of 1,3-butadiene in the area, illustrating both general spatial trends in concentration and the impacts of specific SIS events. At 0.24 ppbv annual average 1,3-butadiene concentration, a group of PSs located near facility fence lines was elevated over a PS group at community/upwind sites (0.07 ppbv). Overall, 1,3-butadiene PS data for S1 and S6 were significantly lower than 24-hour canister data reported in WLATS for similar sites, with the former representing 100% temporal coverage compared to < 9% on average for the latter. This difference indicates that generally lower air shed concentrations have potentially been achieved, likely through a combination of both a lower mobile source signal (from fleet trends) and lower emissions from local industry. Comparison of 1,3-butadiene PS data from this project with BACs is beyond the scope of this paper but is the subject of future work that also explores other PS-measured compounds from this deployment.

In the example emissions event SIS1 in February 2018, a 1.18 ppbv two-week average PS reading was caused by a multiday emission from a yet to be identified, non-facility source very close to S1. During the elevated PS sampling period 20 in June 2018, the airshed was more broadly impacted with PS concentrations ranging from 0.71 ppbv for the near-facility group to 0.46 ppbv for downwind PSs in the community. This case was likely influenced by a known emission event at an industrial facility that was observed from both S1 and S8. For both SIS case studies, time-resolved NGEM approaches yielded valuable supporting data. The lower cost, open-source SPod class of non-speciating FSs in development by EPA provided wind and pollutant data, informing both atmospheric conditions and the direction origin of emissions. Although not the focus of this paper, the potential utility of FS-triggered ACGS to confirm source speciation and instantaneous pollutant concentration levels was illustrated. With significant challenges remaining, high-performance speciated measurements from lower-cost GC equipment showed promise in this initial analysis and provided important pollutant concentration and speciation data for the SIS events described.

Emerging forms of inverse source location modeling were also discussed in the context of the two case studies that affected the local air shed to varying degrees. With a robust time-resolved NGEM signal, iterative optimization schemes to fine-tune source location using combinations of approaches like SDI, BTMs, TCTA, and QUIC can be envisioned. In a similar manner to diagnosing detected emissions, NGEM and SIS models can also be used to document the absence of emissions from known potential source locations as part of envisioned future compliance procedures.

In conclusion, traditional measures of air toxics like 1,3-butadiene in monitoring applications such as EPA’s National Air Toxics Trends Station (NATTS) Network provide valuable long-term exposure information, but limited near-source diagnostic power, due to both low temporal resolution and low measurement density. For example, the closest NATTS to Rubbertown is 150 miles away from Louisville. Similarly, traditional SIS emission estimation approaches are based on relatively infrequent fugitive leak inspections and engineering estimates of malfunction states. Emerging NGEM approaches with a range of measurement performance and implementation cost points can be applied in more places and provide new forms of information to help understand and manage emissions and improve knowledge of impacts to near source communities. NGEM efforts in Rubbertown will continue into 2019 and expand into evaluating odor compounds and community generated (citizen science) odor data.

## Figures and Tables

**Table 1:** Comparison of 1,3-butadiene PS results from this project with approximate WLATS results.

Project	Year	Site	N	Average (ppbv)	Median (ppbv)	σ (ppbv)	Max (ppbv)
NGEM	2017/2018	S1	26	0.15	0.09	0.23	1.18
NGEM	2017/2018	S1 ^1^	25	0.11	0.08	0.09	0.44 ^2^
WLATS	2001	A (S1)	4	0.86	0.54	0.92	2.19
WLATS	2002	A (S1)	30	1.03	0.59	1.24	4.81
WLATS	2003	A (S1)	34	1.53	0.71	1.94	6.62
WLATS	2004	A (S1)	29	0.73	0.16	1.22	5.28
WLATS	2005	A (S1)	31	1.05	0.79	1.16	4.69
NGEM	2017/2018	S6	26	0.07	0.05	0.09	0.46^2^
WLATS	2001	F(S6)	6	0.40	0.42	0.23	0.72
WLATS	2002	F(S6)	31	0.81	0.33	1.30	7.01
WLATS	2003	F(S6)	38	0.72	0.23	1.10	5.09
WLATS	2004	F(S6)	29	0.42	0.16	0.54	2.18
WLATS	2005	F(S6)	31	1.00	0.31	1.57	7.34

^1^ PS values at S1 excluding PS sampling period 12 containing example SIS1 emission event. ^2^ Occurred during PS period 20 containing example SIS2 emission event.

## Supplementary Materials

The following are available online at https://zenodo.org/record/3244802#.XQHoPr6YOpo, Table SM1: Site and deployment details; Table SM2: Summary of 1,3-butadiene PS results; Video SM1: TCTA video for beginning of 2/25/2018 SIS1 event; Video SM2: QUIC model video for beginning of February SIS1 event; Video SM3: TCTA video for 5/7/18 SIS1 event; Video SM4: TCTA video for 6/19/2018 SIS2 event from S1; Video SM5: QUIC model video for SIS2a and SIS2b’ on 6/19/2018; Figure SM1: Area wind roses; Figure SM2: Comparison of field and duplicate PSs; Figure SM3: 1,3-butadiene results by distance and site; Figure SM4: Comparison of S1 SPod and S1 10m wind data for SIS1 event; Figure SM5: Ten second time-resolution S1 SPod wind data for SIS1 event; Figure SM6: Example and description of EnviroSuite BTM; Figure SM7: Example of TCTA for the 2/25/2018 to 2/26/2018 SIS1 event; Figure SM8: View of S1 showing structures affecting plume transport; Figure SM9: SDI plots for February and May 2018 SIS1 events; Figure SM10: Example TCTA for 5/7/2018 SIS1 event; Figure SM11: Comparison of S8 SPod and S1 10m wind data for SIS2 event; Figure SM12: Example of TCTA for 6/19/2018 SIS2 event from S1; Figure SM13: QUIC model of SIS2a and non-optimized location SIS2b.

## Acronyms and Abbreviations

ACGS	automated canister grab sample
BAC	benchmark ambient concentration
BTM	back trajectory model
cts	counts
EPA	United States Environmental Protection Agency
FS	fenceline sensor
FTC	Firearms Training Center
GC	gas chromatograph
HAP	hazardous air pollutant
Hz	hertz (rate of once per second)
KY	Kentucky
km	kilometer
LMAPCD	City of Louisville Metro Air Pollution Control District
MCGS	manually activated canister grab sample
m	meters
m/s	meters per second
MDL	method detection limit
N	sample number
NATTS	National Air Toxics Trends Station
NGEM	next generation emissions measurement
ORD	Office of Research and Development
PID	photoionization detector
PN	part number
ppbv	part(s) per billion by volume
PS	passive sampler
QA	quality assurance
QUIC	Quick Urban & Industrial Complex
SD	secure digital
SDI	source direction indicator
s	standard deviation
SIS	stochastic industrial source
STAR	Strategic Toxic Air Reduction
SM	Supplementary Materials
TCTA	temporally combined trajectory analysis
TOF	time-of-flight
TO	toxic organics
μg/m^3^	micrograms per cubic meter
USD	United States dollar
VOC	volatile organic compound
WLATS	West Louisville Air Toxics Study
